# Selection for narrow gate of emergence results in correlated sex-specific changes in life history of *Drosophila melanogaster*

**DOI:** 10.1242/bio.20147906

**Published:** 2014-06-20

**Authors:** Vishwanath Varma, Nisha N. Kannan, Vijay Kumar Sharma

**Affiliations:** Chronobiology Laboratory, Evolutionary and Organismal Biology Unit, Jawaharlal Nehru Centre for Advanced Scientific Research, Jakkur, Bangalore 560 064, India

**Keywords:** Circadian, *Drosophila*, Precision, Selection, Development time, Lifespan, Fecundity

## Abstract

Since the ability to time rhythmic behaviours in accordance with cyclic environments is likely to confer adaptive advantage to organisms, the underlying clocks are believed to be selected for stability in timekeeping over evolutionary time scales. Here we report the results of a study aimed at assessing fitness consequences of a long-term laboratory selection for tighter circadian organisation using fruit fly *Drosophila melanogaster* populations. We selected flies emerging in a narrow window of 1 h in the morning for several generations and assayed their life history traits such as pre-adult development time, survivorship, adult lifespan and lifetime fecundity. We chose flies emerging during the selection window (in the morning) and another window (in the evening) to represent adaptive and non-adaptive phenotypes, respectively, and examined the correlation of emergence time with adult fitness traits. Adult lifespan of males from the selected populations does not differ from the controls, whereas females from the selected populations have significantly shorter lifespan and produce more eggs during their mid-life compared to the controls. Although there is no difference in the lifespan of males of the selected populations, whether they emerge in morning or evening window, morning emerging females live slightly shorter and lay more eggs during the mid-life stage compared to those emerging in the evening. Interestingly, such a time of emergence dependent difference in fitness is not seen in flies from the control populations. These results, therefore, suggest reduced lifespan and enhanced mid-life reproductive output in females selected for narrow gate of emergence, and a sex-dependent genetic correlation between the timing of emergence and key fitness traits in these populations.

## INTRODUCTION

Circadian clocks have evolved in response to cyclic changes in their environment caused by the rotation of Earth about its own axis. Such cyclic conditions are thought to act as selection pressure for the evolution and subsequent maintenance of circadian clocks (Pittendrigh, 1993; Sharma, 2003; Vaze and Sharma, 2013). The timing of various rhythmic behaviours is thought to be such that it minimises adverse effects of environmental factors and maximises access to resources (Aschoff, 1967; Cloudsley-Thompson, 1960; Fleury et al., 2000; Pittendrigh, 1993). The stability of circadian timekeeping in the face of fluctuating internal as well as external environments is likely to confer organisms with adaptive advantages (Daan, 2000). Therefore, precision of circadian clocks, which is key to their functioning as reliable timekeepers, is believed to be under the influence of selection pressures of the environment (Sharma and Chandrashekaran, 1999; Clodong et al., 2007).

We know a great deal about the mechanisms underlying circadian rhythms and their phase-resetting by light (Dunlap and Loros, 2004; Aronson et al., 1994; Hardin et al., 1990; Zeng et al., 1996; Hunter-Ensor et al., 1996), although little is known about the molecular-genetic bases of clock precision. Since accuracy or precision of circadian clocks in assessing time in the local environment is likely to be closely related to fitness, it is critical to study its genetic variability in natural populations. Previous studies on wild-type animals with different clock periods reported a correlation between precision and circadian period (Pittendrigh and Daan, 1976; Sharma and Chandrashekaran, 1999); however, such a correlation was not seen in mutant strains of animals with distinctly different periods (Bittman, 2012).

Coupling between less precise neuronal oscillators comprising circadian pacemakers produces rhythms with enhanced precision (Enright, 1980; Herzog et al., 2004; Liu et al., 1997). Individual isolated suprachiasmatic nucleus (SCN; the site of central circadian pacemakers in mammals) neurons show low amplitude circadian rhythmicity and higher cycle-to-cycle variability than the whole SCN (Webb et al., 2009). Vasoactive intestinal peptide (VIP) and pigment dispersing factor (PDF), are major coupling agents between the neuronal oscillators in mice and fruit flies, respectively (Ciarleglio et al., 2009; Peng et al., 2003). Loss of these neuropeptides results in asynchronous cellular oscillations and weak behavioural rhythmicity (Liu et al., 1997; Aton et al., 2005). Mice carrying a mutant form of Neuropeptide Y (NPY; a signalling molecule in the non-photic circadian input pathways of mice) show lower inter-individual variability in their period than the wild-type controls (Harrington et al., 2007). Nevertheless, natural variation in clock precision has not been examined for heritable genetic variation upon which selection may act. It would be interesting to examine whether the ability to maintain specific timings for rhythmic behaviours is correlated with life history traits.

In large populations at equilibrium, traits directly related to fitness such as growth rates, adult lifespan and fecundity, bear negative genetic correlations (trade-offs) with one another (Roff, 1996). Such trade-offs are interpreted to be due to pleiotropic alleles that influence two or more components of fitness (Rose and Charlesworth, 1981a). While positively pleiotropic alleles either get quickly fixed (if they increase fitness of both traits) or lost (if they are deleterious), antagonistically pleiotropic alleles persist at intermediate frequencies in a population under the influence of balancing selection (Connallon and Clark, 2013; Barton and Keightley, 2002). Therefore, correlated response in fitness traits to selection for clock precision is likely to suggest the existence of pleiotropic effects of genes influencing both traits (Reznick, 1985), and negative correlations would indicate the cost of possessing stable clocks on fitness (Roff, 1996).

In insects, the duration of pre-adult development and circadian cycle are reported to be positively correlated in clock mutants (Kyriacou et al., 1990) and in wild-type fruit flies (Kumar et al., 2006; Yadav and Sharma, 2013; Takahashi et al., 2013), suggesting the role of an interaction between circadian clocks and the developmental states of flies in timing pre-adult developmental events (Saunders, 2002).

Circadian clocks have also been implicated in the regulation of reproductive fitness in *D. melanogaster*; males carrying a loss of function mutation in the core clock gene *period* (*per*) release fewer sperms causing reduction in the fecundity of females (Beaver et al., 2002; Beaver et al., 2003). However, expression of *per* in the clock neurons of *per*^0^ flies failed to rescue the reduction in egg-output, suggesting a non-circadian function of the *per* gene. Nevertheless, we should be cautious in drawing inferences from studies on life history traits in inbred populations, as they often yield spurious correlations between traits (Rose and Charlesworth, 1981a). In a study on a wild-type strain of *D. melanogaster*, lifespan of flies exposed to Light–Dark (LD) cycles of 24 h period was found to be greater than those maintained under LD cycles of non-24 h periodicities (due to a phenomenon commonly referred as circadian resonance) or constant light (LL, where most circadian behaviours of wild-type flies become arrhythmic) (Pittendrigh and Minis, 1972; von Saint Paul and Aschoff, 1978). Rhythmic wild-type flies live significantly longer than arrhythmic ones (Kumar et al., 2005), and mutants with periods distinctly different from 24 h, showed reduced adult lifespan compared to the wild-type flies, even under LD cycles of period close to their intrinsic period (Klarsfeld and Rouyer, 1998). Under LL, lifespan of flies was shorter than that in LD or DD, although flies laid more eggs in LL than the other two regimes (Sheeba et al., 2000). Overall, organisms reared under resonating (24 h) LD cycles live longer than those maintained in non-resonating (non-24 h) LD cycles or LL (Pittendrigh and Minis, 1972; von Saint Paul and Aschoff, 1978). Moreover, there seems to be some fitness advantage for being rhythmic in terms of extended lifespan, although this cannot be generalised for overall fitness. Although previous studies suggest the role of circadian clocks in the regulation of fitness of organisms, the genetic basis for such phenotypic correlations is still unclear.

In the present study, we compared fitness of fly populations, which were subjected to stabilising selection for narrow gate of adult emergence to assess correlated responses to selection on life history traits. Four replicate populations, derived from four control populations, were subjected to selection for emergence in a narrow window of 1 h close to the peak of daily adult emergence (Kannan et al., 2012). Such stabilising selection for narrow window of adult emergence resulted in an increase in emergence during the selection window and in a reduction in the gate-width of emergence, and in decreased intra- and inter-individual variations in the period of activity/rest rhythm (Kannan et al., 2012).

A classical definition of stabilising selection is that individuals with phenotypes closer to the mean should have greater fitness compared to those constituting the extremes (Travis, 1989). Stabilising selection acts against mutations that produce deleterious alleles which result in deviation in the phenotype from the trait mean and reduce fitness, while balancing selection acts on antagonistically pleiotropic alleles which persist in the population at intermediate frequency (Barton, 1990; Barton and Keightley, 2002). Both means of selection are likely to result in lower fitness for the extreme phenotypes, which in the context of the present study would be those individuals that emerge outside the selection window. Therefore, correlated response to selection for narrow window of emergence would provide evidence for mechanisms that influence both timing of emergence as well as life history traits.

We assayed pre-adult (development time and pre-adult survivorship) and adult (lifespan and lifetime fecundity) fitness traits of fly populations subjected to selection for narrow gate of emergence to examine their fitness, and asked if a correlation exists between the timing of emergence and these life history traits. We chose flies emerging within the selection window in the morning (henceforth the morning window) to represent the adaptive mean phenotype, and flies emerging in an evening window (henceforth the evening window) to represent the extreme non-adaptive phenotype, to examine correlations that may provide evidence for stabilising selection for the mean timing of emergence. We found that inter-individual variance in pre-adult development time was reduced in the selected populations without any cost to its pre-adult fitness. Mated males from the selected populations live as long as the controls, while mated females have significantly shorter lifespan and higher mid-life egg output compared to the controls. Morning emerging mated males from the selected populations live as long as their evening emerging counterparts. On the other hand, morning emerging females have reduced adult lifespan and higher mid-life fecundity than those emerging in the evening, suggesting higher reproductive fitness in the morning emerging flies, and a trade-off between reproduction and lifespan. Interestingly, such correlations between life-history traits and timing of adult emergence are not seen in flies from the control populations. These results suggest that stabilising selection for narrow gate of emergence in the morning results in reduced adult lifespan and enhanced reproductive output in females with the morning emerging selected females having greater reproductive fitness than those emerging in the evening.

## RESULTS

### Selection for narrow gate of adult emergence reduces variance in pre-adult development time

**Fig. 1.**
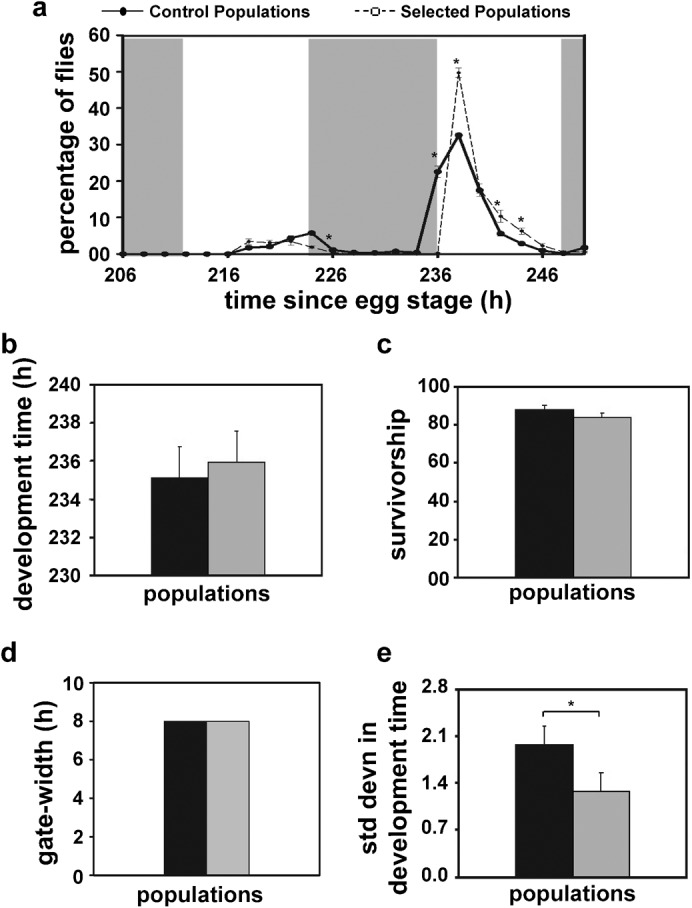
Development time and survivorship of the selected and control flies. (a) Percentage of flies emerging in 2 h windows from the selected and control populations. Light and dark shades represent day-time and night-time, respectively. Time of emergence on the *x*-axis is measured from the time of egg-collection. (b) Mean development time of the selected and control populations. Selected populations show slightly greater development time, although the difference is not statistically significant. (c) Percentage of flies surviving from the egg to adult stage (pre-adult survivorship) is not different between the selected and control populations. (d) Gate-width measured as duration between the onset of emergence (first 2 h window in the day showing greater than 5% emergence) and the offset of emergence (last 2 h window showing greater than 5% emergence) is not different between the selected and control populations. (e) Standard deviation of development time across individual flies is greater in the control populations compared to the selected populations. A total of ∼1000 flies each from the selected and control populations was used in this assay (yielding an overall sample size of *n* = 1969 flies). Error bars are standard errors of mean (SEM). Significant differences of *p*<0.05 from post-hoc comparison are denoted by asterisks. Grey bars indicate the selected populations and black bars indicate the control populations.

To determine the effect of selection for narrow gate of adult emergence on pre-adult fitness traits we assayed pre-adult development time and survivorship of the selected and control populations. The onset of emergence in flies from the selected populations was marginally delayed compared to the controls (Fig. 1a); however, the pre-adult development time (Fig. 1b) and pre-adult survivorship (Fig. 1c) of the selected and control populations did not differ. The gate-width of emergence of the selected and control populations also did not differ under conditions of low larval density (Fig. 1d).

ANOVA on the pre-adult development time data revealed that the effect of genotype (G) was statistically not significant, although ANOVA on inter-individual variance in development time showed a statistically significant effect of G (*F_1,3_* = 29.73; *p*<0.01; Fig. 1b,e). Post-hoc multiple comparisons using Tukey's test revealed that although the mean development time of the selected and control populations did not differ, selected flies showed reduced variation in development time compared to the controls (Fig. 1). These results suggest that flies selected for narrow gate of emergence do not differ in their pre-adult development time, but have become more coherent in their emergence time compared to the controls without incurring any cost to their pre-adult fitness.

### Females from the selected populations live shorter than the controls and show time of emergence dependent difference in lifespan

**Fig. 2.**
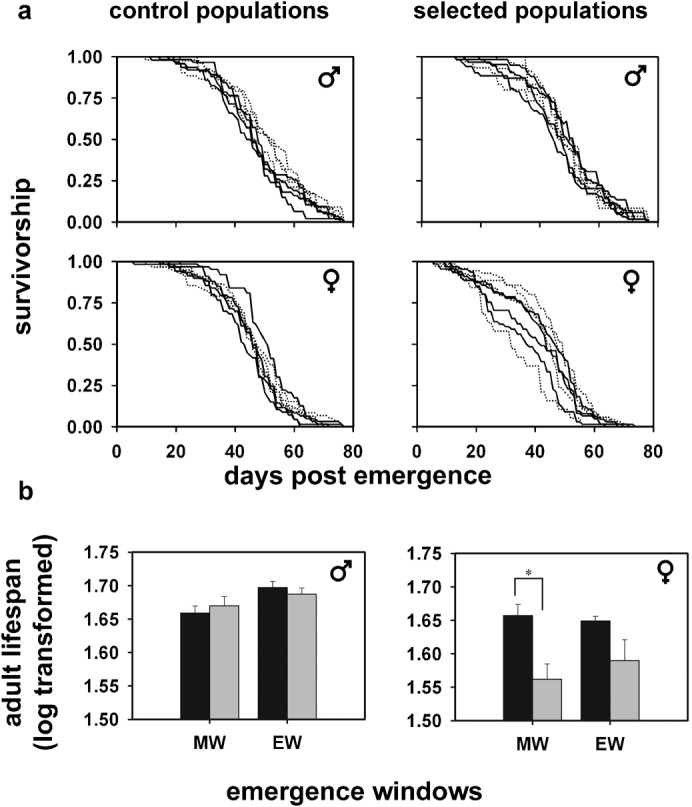
Survivorship curves and adult lifespan of mated males and females from the selected and control populations. (a) Relative percentage of flies survived on the *y*-axis plotted against the age of flies in days post emergence on the *x*-axis. Left and right panels show survivorship curves of the control and selected populations, respectively, in this section while top and bottom rows represent male and female flies, respectively. Each panel compares the survivorship of flies emerging in the morning with those emerging in the evening window. Continuous lines represent morning emerging (ZT01–02, M window) flies whereas dashed lines represent evening emerging (ZT10–11, E window) flies. Morning emerging mated females from the selected populations have reduced adult lifespan compared to their controls. (b) Comparisons of the mean log transformed adult lifespan data between the selected and control populations of the morning and evening emerging flies. Left and right panels show adult lifespan data of male and female flies, respectively, in this section alone. Emergence window is indicated on the *x*-axis as morning (M) and evening (E) windows. Error bars are SEM. Significant differences of *p*<0.05 from post-hoc comparison are denoted by asterisks. Grey bars indicate the selected populations and black bars indicate the control populations. About 200 flies each from the morning and evening emerging males and females from the selected and control populations were used for this assay (yielding an overall sample size of *n* = 1739).

We next compared mean lifespan, which is an important adult fitness trait, in mated flies from both selected and control populations. We chose flies emerging within a narrow window of 1 h each in the morning and evening as representatives of adaptive and non-adaptive phenotypes, respectively, since our selection regime is such that only flies emerging in the morning window contribute to the next generation. Hence, flies were selected from the morning window (Zeitgeber Time 01–02, where time of lights coming on under 12:12 h LD cycles is considered as ZT00 and lights-off as ZT12) and evening window (ZT10–11). We found that males, irrespective of the time of emergence, lived longer than females both in the selected and control populations. ANOVA revealed statistically significant effects of sex (S) (*F_1,1707_* = 23.72, *p*<0.05) and genotype (G) (*F_1,1707_* = 10.78, *p*<0.05). Post-hoc multiple comparisons using Tukey's test revealed that morning emerging mated females from the selected populations lived significantly shorter than morning emerging controls. Post-hoc multiple comparisons also revealed that in the selected populations, adult lifespan of morning and evening emerging males did not differ. Morning emerging females from the selected populations lived shorter than those emerging in the evening, although this difference was not statistically significant (Fig. 2a,b). Such a time of emergence dependent difference in adult lifespan was not seen in the control flies. These results suggest reduced lifespan in mated females from the selected populations and a link between timing of emergence and adult lifespan resulting in a sex-specific correlated response to selection for narrow gate of emergence.

### Morning emerging flies from the selected populations have enhanced mid-life fecundity

**Fig. 3.**
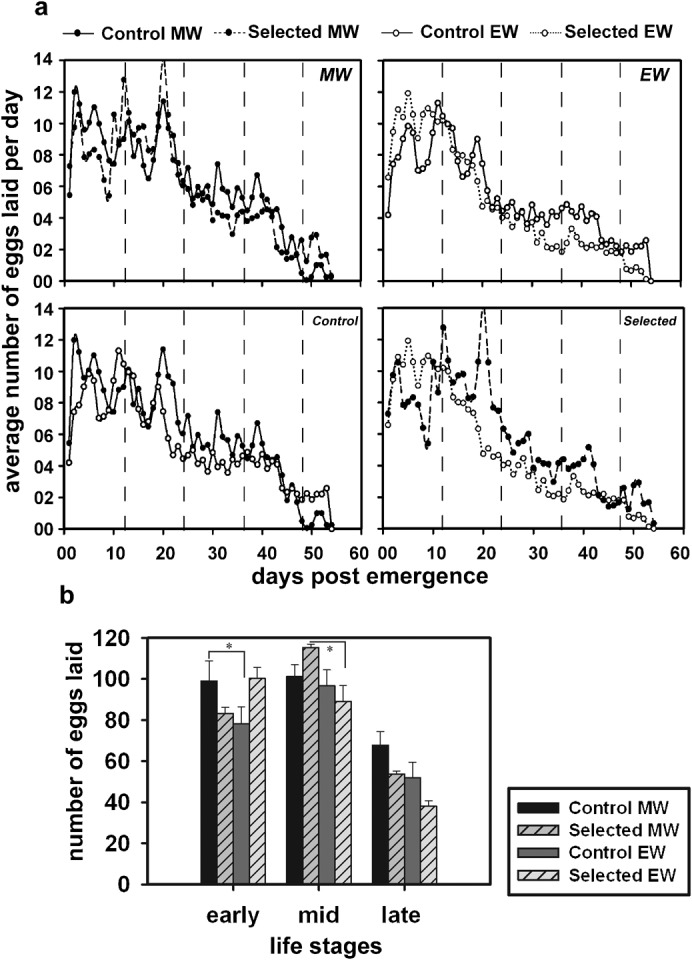
Lifetime fecundity and fecundity across age blocks in flies from the selected and control populations emerging in the morning and evening windows. (a) Top panels show comparisons of lifetime fecundity between the selected and control flies emerging in the same window whereas bottom panels show comparisons across the emergence windows. The number of eggs laid on a particular day averaged across individuals is plotted against the age of the fly measured as days after emergence. The dotted lines represent the divisions of the lifetime into relevant age blocks, which are then pooled and analysed in the bottom-most panel. (b) Comparison of fecundity of the selected and control flies across early (1–12), mid (13–24) and late (25–36) life stages. The very late last stage was excluded since very few flies survived until 48 days. The sample sizes of flies that survived until the age of 36, and were thus used for analyses were *n* = 43 and *n* = 47 for the morning and evening emerging windows, respectively, for the selected populations, and *n* = 49 and *n* = 48 for the morning and evening emerging windows, respectively, for the control populations. A total of ∼45 females each from the morning and evening emerging flies from the selected and control populations was used (yielding an overall sample size of *n* = 187). Error bars are SEM. Significant differences of *p*<0.05 from post-hoc comparisons are denoted by asterisks.

We also assayed daily fecundity across the adult lifespan of flies by counting the number of eggs laid by individual females every day, from the day of emergence until death (Fig. 3a,b). The daily fecundity of these flies is around 10 eggs per day, which is on the lower side for outbred populations of *D. melanogaster* (Fig. 3a). One of the reasons for this low fecundity could be the smaller body size of these females owing to their rearing under crowded larval conditions. Repeated measures ANOVA on the lifetime fecundity data with window of emergence (W) and genotype (G) as fixed factors and fecundity across age blocks (A) as repeated measure, revealed statistically significant effects of W (*F_1,183_* = 6.07, *p*<0.01), A (*F_2,366_* = 121.35, *p*<0.001) and G×A (*F_2,366_* = 4.72, *p*<0.009) and W×G×A interactions (*F_2,366_* = 10.87, *p*<0.001). The fly populations used in our study have been maintained on a 21 day generation cycle, which requires them to lay eggs on the 12th day after emergence to contribute to the next generation. Therefore, we divided the stages of adult life of flies into blocks of 12 days each, with early (days 1–12), mid (days 13–24) and late life stages (days 25–36). Only flies that survived and laid eggs until the age of 36 days were considered for the analysis. Post-hoc multiple comparisons using Tukey's test revealed that the total egg output of the selected and control flies did not differ statistically; however, there was a trend of lower early-life fecundity and greater mid-life fecundity in the selected populations compared to the controls. Post-hoc multiple comparisons on the early-life fecundity (days 1–12) data revealed that flies from the control populations emerging in the morning had greater fecundity than those emerging in the evening (Fig. 3b). This trend was reversed in the selected populations, although the difference was statistically not significant.

Mid-life fecundity (days 13–24) of the morning emerging flies from the selected populations was significantly greater than that of those emerging in the evening, whereas mid-life fecundity of the morning and evening emerging flies from the control populations did not differ (Fig. 3b). Mid-life fecundity of the selected populations was also greater than that of the controls, although this difference was statistically significant. Hence, the correlation of time of emergence with early-life fecundity, seen in the controls, is broken in the selected populations as a result of selection. Instead, the lower early-life fecundity in the morning emerging flies from the selected populations was compensated by an increase in their mid-life fecundity. The late-life fecundity was reduced in the evening emerging flies (compared to morning emerging flies) across both the populations. These trends were consistently seen even when the age of the flies was divided into 7 or 10 day age blocks. However, since we excluded those flies that did not lay eggs even though they were alive for 36 days, there was negligible increase in sample size using these smaller age blocks; hence, we persisted with the analysis on 12 day age blocks. Thus, the results of our assays revealed that although selected flies did not show differences in their total egg output, they displayed an age dependent enhancement in reproductive output during their mid-life stage. It also demonstrates the effects of selection for narrow window of emergence on egg output across different life stages of flies, which provide evidence of a trade-off between early and mid-life fecundity in the morning emerging flies from the selected populations.

## DISCUSSION

Correlations between life history traits suggest a common genetic architecture or somatic cost of certain traits on others. Correlated responses to selection further affirm that genetic variation upon which selection acts on a particular trait also includes pleiotropic effects on other traits. The occurrence of such pleiotropic effects may be due to functional relatedness among the traits. Although there is evidence of the role of circadian clocks in the regulation of development time (Kyriacou et al., 1990; Miyatake, 1997; Miyatake, 2002; Kumar et al., 2005) and adult lifespan (Pittendrigh and Minis, 1972; Klarsfeld and Rouyer, 1998; Hendricks et al., 2003), it is still unclear whether such effects are solely based on phenotypes or if genetic causation can be attributed.

The results of our studies suggest that stabilising selection for narrow gate of emergence in the morning does not affect pre-adult development time and survivorship. Although we observed no difference in gate-width between the selected and control populations, it must be noted that the development time assay was done under uncrowded conditions (30 eggs per vial) in contrast to the normal maintenance conditions for the populations where the larval density is about 300 eggs per vial. In the crowded maintenance conditions, the gate-width of emergence of the selected populations has evolved to be significantly shorter than the controls as a direct response to selection (Kannan et al., 2012). Such differences in the expression of response to selection in assay environments different from the maintenance environment have also been reported previously (Ackermann et al., 2001). However, despite the absence of differences in their mean development time, flies from the selected populations showed lower inter-individual variance in development time. This results in greater coherence in emergence time in selected populations.

Moreover, circadian period and development time are usually positively correlated, with short period individuals developing faster than those with long period (Kyriacou et al., 1990; Yadav and Sharma, 2013; Miyatake, 1997), therefore, the lack of change in the mean development time of the selected populations (Fig. 1b) despite shortening of period (Kannan et al., 2012) is counterintuitive. This observation can be partly explained by the fact that flies from the selected populations show enhanced synchrony and reduced gate-width of emergence, due to strengthened effect of emergence gate, which may prevent flies from emerging outside the morning selection window even if they are developmentally mature (Mukherjee et al., 2012). This is consistent with an overall increase in precision of both emergence and activity/rest rhythms in the selected populations (Kannan et al., 2012).

Mated males from the selected populations live as long as the controls, whereas mated females from the selected populations have significantly reduced adult lifespan compared to the controls. Thus, there appears to be a sex-specific evolution of reduced lifespan in the females of the selected populations. There are reports of sexual dimorphism in ageing and mortality in several species with males usually being shorter lived than females (Owens, 2002; Clutton-Brock et al., 1985; Promislow and Harvey, 1990) due to factors such as competition, physiological costs of sex hormones and high-risk, high-return reproductive strategies (Trivers, 1972; Vinogradov, 1998). However, empirical studies confirm that such male biased mortality is not universal across species due to factors such as selection pressure, increased male mating success with age and variable sex roles (Bonduriansky et al., 2008; Promislow, 2003). Since our assay conditions presumably do not result in intense male competition, we do not see a reduction in male lifespan (Fig. 2). Additionally, it is known that artificial selection can result in sex-specific responses (Winkler et al., 2012; Hoffmann et al., 2005). Our observations are consistent with the fact that quantitative trait loci for longevity in *D. melanogaster* show sex-specific effects on lifespan (Nuzhdin et al., 1997).

Although adult lifespan of morning and evening emerging males from the selected populations does not differ, morning emerging females live shorter than their evening emerging counterparts (Fig. 2). Interestingly, flies from the control populations do not show such time of emergence dependent difference in adult lifespan. This reduction in lifespan of the morning emerging females from the selected populations can be attributed to mating and reproductive costs on survival since this lower lifespan is compensated by greater mid-life fecundity in these flies (Fig. 3). These results suggest that morning emergence is correlated with greater mid-life fecundity around the day of egg-collection in the maintenance regime of these populations, and has a fitness advantage for flies from the selected populations in terms of becoming a part of the breeding pool for the next generation. This enhancement of egg output around the day of egg collection has been reported in studies where early or late fecundity has been selected for in fly populations (Rose and Charlesworth, 1981b). This may be due to age-specific genetic variance in fecundity (Tatar et al., 1996; Leips et al., 2006). Lower adult lifespan and higher mid-life fecundity in females from the selected populations emerging in the selection window as a correlated response to selection for narrow gate of emergence can be taken as evidence of antagonistically pleiotropic effects or trade-offs between these traits (Reznick, 1985). This evolution of enhanced egg production at the cost of adult lifespan in females is noteworthy but nevertheless understandable, given the importance of fecundity in female reproductive fitness. Such sex-specific effects on reproductive trade-offs have been previously reported in natural populations of crickets (Zajitschek et al., 2009). Thus, stabilising selection on narrow window of emergence enhances mid-life fecundity of flies, although at the cost of reduced adult lifespan.

Morning emerging flies from both the populations show greater late-life fecundity relative to those emerging in the evening. Late-life fecundity plateaus in females, which lay fewer eggs early in their life (Rose and Charlesworth, 1981a). However, in controls, the morning emerging flies not only have higher early-life fecundity, they also lay more eggs during their late-life stage compared to the evening emerging flies. On the other hand, morning emerging flies from the selected populations compromised their early-life fecundity for greater mid-life fecundity. These results are consistent with the notion of trade-off in reproductive efforts between the successive life stages (Williams, 1966; Gadgil and Bossert, 1970), which predicts that females that lay fewer eggs early in their life, live longer and lay more eggs later in their life (Rauser et al., 2003). Such negative correlations between fecundity at different stages of life, and between high fecundity and adult lifespan have been reported earlier in *Drosophila* (Rose and Charlesworth, 1981a; Rose and Charlesworth, 1981b). Thus, selection for narrow window of emergence yields morning emerging females with greater mid-life fecundity at the cost of reduced early-life fecundity and adult lifespan, consistent with the expectation of trade-offs between life-history traits.

Morning emerging mated females have higher mid-life fecundity than those emerging in the evening, which is consistent with the fact that evening emergence is maladaptive in flies from the selected populations. Since in these fly populations, emergence in the morning is strictly selected for, the proportion of flies emerging in the morning is much greater than those emerging in the evening (Kannan et al., 2012). Thus, although fecundity of selected populations is reduced compared to the controls at late-life stage, under the given protocol of a 21 day generation cycle, flies from the selected populations would have an adaptive advantage in terms of survival and egg output on the day when it matters the most. Hence, we can conclude that female flies selected for narrow gate of emergence have greater reproductive fitness than the controls under the maintenance regime. However, we have only considered flies with mean and extreme phenotypes of emergence timing and compared their life history traits, while conclusions regarding overall fitness of a population should be based on a more random sampling of flies from the population. Since a great majority of the flies emerge around the morning window of selection; our conclusions are likely to be robust for flies emerging across the day.

In summary, the results of our study revealed reduction in the variance in development time at no cost to pre-adult fitness. Adult lifespan of females from the selected populations is shorter compared to the controls. However, this reduction of lifespan in females is compensated by a concurrent increase in their mid-life fecundity. Flies from the selected populations show time of emergence dependent difference in adult fitness, albeit in a sex-specific manner. Morning emerging females from the selected populations live shorter than their evening emerging counterparts. Morning emerging females from the selected populations lay more eggs at the mid-life stage suggesting enhanced reproductive fitness under the maintenance regime. Interestingly, such time-of-emergence based differences in adult fitness traits are not seen in the controls. Thus, we find evidence of enhanced age-specific reproductive output in females from the selected populations for emergence in the morning compared to evening. We interpret these results as evidence of genetic correlations between timing of emergence and life history traits, which indicate adaptive significance of enhanced clock precision in these flies.

## MATERIALS AND METHODS

### Stock maintenance and standardisation

The populations used in the present study were derived from four ancestral baseline populations of *D. melanogaster* that have been maintained in the laboratory for several hundred generations under 12:12 h light/dark cycles (LD) at 25°C on banana-jaggery (BJ) food (Sheeba et al., 1998). Four precision populations were initiated by selecting for flies that emerged during Zeitgeber Time 01–02 (ZT01–02), where time of lights coming on under 12:12 h LD cycles is considered as ZT00 and lights-off as ZT12. Four control populations were also initiated along with the selected populations in which flies emerging throughout the day were used. Therefore, the control populations experienced all conditions similar to the selected populations except that they were not under any conscious selection for the timing of emergence. Flies emerging over four successive days (9–12th day after egg collection) were collected to form the breeding pool for the next generation. A total of 1200 adults per population, with approximately equal number of males and females, was maintained in plexiglass cages of 25×20×15 cm^3^ dimension with BJ medium. Flies were fed with yeast–acetic-acid paste for 3 days before egg collection to induce egg production. Three days later, eggs were collected over a 3 h window on BJ medium and approximately ∼300 eggs were transferred into glass vials (18 cm height × 2.4 cm diameter) containing ∼10 ml of BJ medium. Exactly 48 and 16 such vials were set-up in every generation for each of the selected and control populations, respectively. Both selected and control populations were maintained on a 21 day discrete (non-overlapping) generation cycle. To minimise non-genetic parental effects, which may have been caused by the imposition of the selection protocol, prior to all our assays, the selected and control populations were subjected to one generation of common rearing when the selection pressure was relaxed. The progeny of such flies will be henceforth referred to as “standardised flies”.

### Development time and survivorship assays

After 90 generations of selection, the pre-adult development time and survivorship of flies from the selected and control populations were assessed. From each standardised population, eggs laid during a 2 h window (ZT01–03) were collected and exactly 30 eggs were dispensed into each long vial (18 cm height × 2.4 cm diameter) containing ∼6 ml BJ medium. For the assays, ten such vials from each replicate population were introduced into cyclic LD condition created inside an incubator (Percival, Perry, IA, USA). Temperature (25±1°C) and humidity (75±5%) inside the incubator were monitored throughout the study and were found to be stable. Fluorescent white light of intensity ∼100 lux was used during the light phase and dim red light of wavelength greater than 650 nm was used during the dark phase of LD cycles. About 1200 eggs each of the selected and control populations were dispensed into glass vials with 30 eggs per vial, out of which a total of ∼1000 flies emerged as adults for selected and control populations and their development times were recorded, yielding an overall sample size of *n* = 1969 flies. Vials containing eggs were monitored daily for darkened pupae and thereafter every 2 h for emerging adults. To estimate pre-adult development time and survivorship, adults were collected every 2 h and counted. The development time of a fly, in hours, was calculated as the time interval between the midpoint of 2 h egg collection window and the mid-point of 2 h period during which the fly emerged as adult. For the analysis on time of emergence, percentage of flies emerging every 2 h was used. Pre-adult survivorship was estimated as the fraction of eggs, in each vial, that successfully developed and emerged as adults. The gate-width of emergence was taken as duration between the onset of emergence (first 2 h window in the day showing greater than 5% emergence) and the offset of emergence (last 2 h window showing greater than 5% emergence). The threshold of 5% has been used as a standard cut-off for measuring gate-width in previous studies on these populations as well as other flies (Kannan et al., 2012; Prabhakaran et al., 2013).

### Adult lifespan assay

Adult lifespan of flies from the selected and control populations was assessed after 100 generations of selection. From the standardised populations of selected and control flies, eggs laid over a 2 h window on BJ medium were collected. From each replicate population, ∼300 eggs were transferred into glass vials (18 cm height × 2.4 cm diameter) containing 10 ml of BJ medium. For each population, 24 such vials were maintained under LD cycles until the adults emerged. From the standardised populations we collected flies, which emerged during the selection (ZT01–02: morning – M) and evening windows (ZT10–11: evening – E) to represent the mean and extreme emergence phenotypes, respectively. For the adult lifespan assay of mated flies, 4 males and 4 females were introduced in each vial and adult lifespan of twenty such vials for each replicate population were monitored until all flies in all the vials died. Flies were provided with fresh BJ medium every alternate day and vials were checked every day for the death of flies. From the selected populations, 225 morning emerging and 159 evening emerging males, and 248 morning emerging and 183 evening emerging females were used for this assay. Similarly, from the control populations, 221 morning emerging and 206 evening emerging males, and 258 morning emerging and 239 evening emerging females were used, yielding an overall sample size of *n* = 1739 flies.

### Fecundity assay

After 100 generations of selection, fecundity of flies from the selected and control populations was assayed to examine if there was any effect of selection on the reproductive fitness. From the standardised populations we collected flies, which emerged during the morning (ZT01–02) and evening windows (ZT10–11), similar to the lifespan assay. From these two sets of flies (morning and evening emerging), males and females were introduced in pairs into glass vials containing ∼3 ml of BJ food. Twenty such vials from each population were introduced into LD cycles. The number of eggs laid every day post-emergence until the day of death of the female was counted to assess lifetime fecundity of the fly. During the fecundity assay, flies were transferred into fresh food vials every day and the number of eggs laid on the previous day was recorded. The duration of average lifespan was divided into equal windows of 12 days based on the intervals of age with distinct patterns of egg-laying to compare across selected and control populations and the two emergence windows. Thus the age of females was divided into early (1–12 days), mid (13–24 days) and late (25–36 days) life stages. Since very few flies lived until the late-life stage (37–48 days) and eggs laid at this stage was very low, we did not include this age block in the analyses. Overall, 43 morning emerging and 47 evening emerging females from the selected populations and 49 morning emerging and 48 evening emerging females from the control populations were used, yielding an overall sample size of *n* = 187 flies.

### Statistical analysis

Time of emergence was analysed using mixed model analysis of variance (ANOVA) treating replicate populations as random factor and genotype (G) and timing of emergence (T) as fixed factors. Pre-adult development time and survivorship were analysed separately using mixed model analysis of variance (ANOVA) treating replicate populations as random factor and genotype (G) as fixed factor. For the adult lifespan assay, genotype (G), sex (S) and emergence window (W) were treated as fixed factors. All the analyses for adult lifespan were performed on natural log transformed values of the individual lifespan data, since the adult lifespan data have a long right-hand tail for which mean value is not an appropriate measure for comparison. Repeated measures ANOVA was used for the lifetime fecundity data with emergence window (W) and genotype (G) as fixed factors and daily number of eggs as the repeated measure across age blocks (A). The fecundity data across four replicate populations were pooled after noting that there was no main effect of the replicate populations. Post hoc multiple comparisons were done using Tukey's test. The error bars used in the figures are standard error of mean (SEM). All our analyses were implemented on STATISTICA for Windows Release 5.0 B (1995, StatSoft).
